# Development and psychometric evaluation of a pre-sarcopenia risk assessment scale for sedentary young adults with type 2 diabetes mellitus

**DOI:** 10.3389/fpubh.2026.1960222

**Published:** 2026-10-30

**Authors:** Chen-Xia Zhang, Cong-Cong Jin, Shi-Wei Liu, Xiao-Fang Wei, Jiao-Jun Mu

**Affiliations:** Department of Endocrinology, Shanxi Bethune Hospital, Shanxi Academy of Medical Sciences, Third Hospital of Shanxi Medical University, Tongji Shanxi Hospital, Taiyuan, China

**Keywords:** pre-sarcopenia, psychometric evaluation, risk assessment, risk perception, sedentary young adults, type 2 diabetes mellitus

## Abstract

**Aims:**

The aim of this study was to develop a self-reported risk-assessment scale for pre-sarcopenia-related behavioral and functional risk in sedentary young adults with type 2 diabetes mellitus (T2DM) and to assess its psychometric properties.

**Methods:**

Following COSMIN guidelines, an initial item pool was generated via literature review and semi-structured interviews, then refined through two Delphi rounds and a pilot survey. A convenience sample of 435 patients (June 2024–February 2025) was recruited; 215 participated in exploratory factor analysis (EFA) and 220 in confirmatory factor analysis (CFA). Reliability and validity were evaluated.

**Results:**

The final 18-item scale comprises four dimensions: behavioral risk perception, muscle function monitoring, sedentary behavior characteristics, and intervention intention. EFA extracted four factors explaining 81.25% of the total variance. CFA demonstrated good model fit (*χ*^2^/df = 1.583, RMSEA = 0.076, SRMR = 0.038, CFI = 0.965, GFI = 0.917). The scale-level content validity index was 0.92; Cronbach’s α was 0.894 and split-half reliability 0.792.

**Conclusion:**

The newly developed scale showed satisfactory preliminary reliability and construct validity. It should be regarded as a self-reported risk-assessment instrument for pre-sarcopenia-related behavioral and functional risk, rather than a diagnostic or case-finding tool for pre-sarcopenia. Positive risk identification should be confirmed by objective measures such as handgrip strength, muscle mass, and physical performance.

## 1 Introduction

With rapid urbanization and the widespread adoption of digital technologies, sedentary behavior has become a predominant lifestyle pattern among young adults. Among young adults with type 2 diabetes mellitus (T2DM), prolonged sedentary behavior interacts with underlying metabolic abnormalities, further increasing the risk of impaired muscle health (1, 2). Prior studies have reported that Chinese adults aged 18 to 35 years spend more than 8 h per day engaged in sedentary activities, with the prevalence of sedentary behavior reaching 78.3% (3). Sedentary behavior contributes to the loss of muscle mass and deterioration of muscle function through multiple pathological mechanisms, including the inhibition of muscle protein synthesis, reduced muscle metabolic activity, and the induction of chronic low-grade inflammation, and has been identified as an independent risk factor for pre-sarcopenia (1, 4, 5).

As the early stage of sarcopenia, pre-sarcopenia is characterized by an initial decline in muscle strength and impaired physical performance during activities of daily living among young adults with T2DM who engage in prolonged sedentary behavior (6, 7). Without timely intervention, the risk of progression to sarcopenia within 5 years increases substantially (8). Currently available sarcopenia screening instruments have been developed primarily for older adults, with screening items emphasizing age-related characteristics such as a history of falls and walking difficulties. Consequently, these instruments demonstrate limited sensitivity (52.6%) in detecting sedentary behavior-related muscle dysfunction in young adults (9, 10). In China, existing studies have primarily focused on the assessment of muscle function in athletes and manual laborers, and validated screening instruments specifically designed for sedentary young adults are not available (11, 12).

The development of a risk-assessment instrument tailored to the behavioral and metabolic characteristics of young adults with T2DM who engage in prolonged sedentary behavior and that is capable of identifying early declines in muscle function is of considerable clinical importance for facilitating timely and targeted intervention. In accordance with the COSMIN guidelines, this study integrated the pathological mechanisms of pre-sarcopenia with the characteristics of sedentary behavior to develop a pre-sarcopenia risk assessment scale for sedentary young adults with T2DM and to assess its psychometric properties. The aim of this study was to provide a scientifically reliable and clinically applicable instrument for preliminary risk assessment of pre-sarcopenia-related behavioral and functional risk in this population. The instrument is not intended to replace clinical diagnosis, and positive risk identification requires confirmation with objective measures.

## 2 Materials and methods

### 2.1 Study participants

#### 2.1.1 Participants

Convenience sampling was used to recruit eligible participants from the outpatient clinics and inpatient wards of the Department of Endocrinology at our Hospital between June 2024 and February 2025. The inclusion criteria were as follows: (1) aged 18 to 35 years; (2) diagnosed with T2DM according to the *Chinese Guidelines for the Prevention and Treatment of Type 2 Diabetes Mellitus (2020 Edition)* (13); (3) reporting ≥ 8 h of sedentary behavior per day during waking hours, as assessed using the Sedentary Behavior Assessment Questionnaire; and (4) providing written informed consent and voluntarily participation in the study.

The exclusion criteria were as follows: (1) a confirmed diagnosis of sarcopenia, osteoporosis, thyroid dysfunction, or other diseases that significantly affect muscle metabolism; (2) severe diabetes-related complications; (3) inability to complete the basic muscle function assessment because of physical limitations; (4) regular participation in high-intensity resistance training within the previous 6 months or being a professional athlete; and (5) pregnancy or lactation.

This study was approved by the Ethics Committee of our Hospital, and written informed consent was obtained from all participants prior to study enrollment.

The sample size was determined according to recommendations for factor analysis. For EFA, at least 5–10 participants per item are recommended, and for CFA, a minimum of 200 participants is generally recommended. Because independent samples were used for EFA and CFA, we planned to recruit approximately 200 participants for each analysis. Allowing for an anticipated 10% rate of invalid or incomplete questionnaires, the target sample size was set at approximately 440 participants. A total of 435 participants were finally included (215 for EFA and 220 for CFA). The participants were randomly assigned, using a random number table, to either the EFA group (*n* = 215, 49.4%) or the CFA group (*n* = 220, 50.6%). No statistically significant differences were observed between the two groups regarding baseline characteristics, including age, sex, and duration of diabetes (all *p* > 0.05), indicating that the groups were comparable (Table 1).

**Table 1:** Baseline characteristics of participants in the EFA and CFA groups.

Items	Total sample (*n* = 435)	EFA group (*n* = 215)	CFA group (*n* = 220)	Statistical value	*p*-value
Age (years)	26.5 ± 4.1	26.2 ± 4.3	26.7 ± 3.9	*t* = −1.269	0.205
Sex				*χ*^2^ = 0.128	0.721
Male	208 (47.8%)	105 (48.8%)	103 (46.8%)		
Female	227 (52.2%)	110 (51.2%)	117 (53.2%)		
BMI (kg/m^2^)	22.8 ± 3.5	22.6 ± 3.7	23.0 ± 3.3	*t* = −1.201	0.230
Years of education (years)	16.2 ± 2.8	16.4 ± 2.9	16.0 ± 2.7		
Occupation type				*χ*^2^ = 3.421	0.331
Office staff	150 (34.5%)	72 (33.5%)	78 (35.5%)		
Information technology practitioners	110 (25.3%)	56 (26.0%)	54 (24.5%)		
Students	100 (23.0%)	53 (24.7%)	47 (21.4%)		
Freelancers	45 (10.3%)	20 (9.3%)	25 (11.4%)		
Others	30 (6.9%)	14 (6.5%)	16 (7.3%)		
Diabetes-related indicators					
Diabetes duration (years)	3.2 ± 2.1	3.1 ± 2.2	3.3 ± 2.0	*t* = −0.976	0.330
Glycated hemoglobin (%)	7.5 ± 1.2	7.6 ± 1.3	7.4 ± 1.1	*t* = 1.642	0.101
Fasting blood glucose (mmol/L)	8.2 ± 1.8	8.1 ± 1.7	8.1 ± 1.7	*t* = 1.074	0.284
Sedentary and behavioral indicators					
Daily sedentary time (h)	8.5 ± 1.6	8.4 ± 1.7	8.6 ± 1.5	*t* = −1.302	0.194
Daily moderate-to-vigorous physical activity (min)	26.5 ± 14.8	25.6 ± 15.1	27.3 ± 14.5	*t* = −1.162	0.246
Maximum duration of continuous sedentary time (h)	2.8 ± 0.9	2.7 ± 1.0	2.9 ± 0.8	*t* = −1.891	0.059
Muscle function indicators					
Handgrip strength (kg)	36.8 ± 8.0	36.5 ± 8.2	37.1 ± 7.9	*t* = 1.283	0.200
5-repetition sit-to-stand test (s)	11.1 ± 2.4	11.2 ± 2.5	10.9 ± 2.3	*t* = −1.403	0.161

#### 2.1.2 Consulted experts

Between July and September 2024, experts from tertiary hospitals and universities across seven provinces and municipalities in China were invited to participate in the Delphi consultation. The inclusion criteria for experts were as follows: (1) a bachelor’s degree or higher; (2) an intermediate professional title with ≥ 10 years of relevant professional experience or an associate senior professional title or above with ≥ 5 years of relevant professional experience; (3) professional experience in endocrinology and metabolic diseases, diabetes care, rehabilitation medicine, musculoskeletal health, nursing management, or nursing education; (4) familiarity with the methodology and procedures for scale development; and (5) providing informed consent and voluntary participation.

A total of 19 experts participated in the Delphi consultation, including 7 men and 12 women, with a mean age of 42.1 ± 5.8 years (range, 33–55 years). Regarding educational attainment, 6 experts held bachelor’s degrees, 7 held master’s degrees, and 6 held doctoral degrees. Their professional experience ranged from 6 to 31 years, with a mean of 14.2 ± 6.1 years. The panel comprised of 8 experts with intermediate professional titles, 7 with associate senior professional titles, and 4 with senior professional titles. The areas of expertise included endocrinology (*n* = 5), diabetes specialist nursing (*n* = 4), rehabilitation therapy (*n* = 4), nursing management (*n* = 3), and nursing education (*n* = 3).

### 2.2 Methods

#### 2.2.1 Theoretical framework

This study adopted the Health Belief Model (HBM) as the theoretical framework for scale development (14). The HBM is a widely used theoretical model for explaining and predicting health-related behaviors. According to the HBM, engagement in health behaviors is primarily influenced by six core constructs: perceived susceptibility, perceived severity, perceived benefits, perceived barriers, cues to action, and self-efficacy (15, 16).

The present scale was developed to identify young adults with T2DM who engage in prolonged sedentary behavior and are at risk of pre-sarcopenia. As a preliminary risk-assessment instrument for risk perception and behavioral intention, the scale was conceptually grounded in the HBM. The scale is not intended to replace clinical diagnosis; positive risk identification should be confirmed by objective measures such as handgrip strength, muscle mass, and physical performance. Specifically, the behavioral risk perception dimension reflects perceived susceptibility and perceived severity, whereas the intervention intention dimension corresponds to perceived benefits, cues to action, and self-efficacy. Assessment of these constructs supports the identification of individuals who are more likely to recognize their risk of impaired muscle health and demonstrate a willingness to adopt preventive interventions, thereby supporting earlier identification and intervention for pre-sarcopenia.

#### 2.2.2 Scale development

##### 2.2.2.1 Establishment of research group

The research team comprised of 10 members, including 1 chief endocrinologist, 1 head nurse specializing in diabetes care, 1 rehabilitation therapist, 1 clinical nutrition physician, 1 professor with expertise in research methodology, 2 postgraduate nursing students, and 3 diabetes specialist nurses. Clinical expertise was provided by the endocrinology team throughout the study. The scale items were reviewed by the rehabilitation and clinical nutrition specialists. The methodology team was responsible for study design, literature review, qualitative interviews, and statistical analysis. All team members participated in the Delphi consultation, item revision, and quality control to ensure the scientific rigor and clinical applicability of the scale.

##### 2.2.2.2 Literature retrieval

A comprehensive literature search was conducted using a combination of controlled vocabulary and free-text terms. Chinese search terms included sedentary behavior, sedentary lifestyle, sarcopenia, pre-sarcopenia, T2DM, screening, assessment, and risk prediction. The corresponding English terms included Sedentary Behavior, Sedentary Lifestyle, Sarcopenia, Pre-sarcopenia, Type 2 Diabetes, Screening, Assessment, and Risk Prediction. The databases searched included PubMed, Embase, CINAHL, China National Knowledge Infrastructure, and Wanfang Data, covering the period from database inception to May 2024. A total of 1,862 records were initially identified. Following duplicate removal, title and abstract screening, and full-text review, 22 studies were included. Through iterative discussions and content analysis, the research team identified key concepts related to sedentary behavior, the pathophysiology of pre-sarcopenia, manifestations of muscle dysfunction, and health risk perception were identified by the research team, thereby establishing the preliminary item pool.

##### 2.2.2.3 Semi-structured interviews

Purposive sampling was used to recruit five young adults with T2DM who engaged in prolonged sedentary behavior for pilot interviews between June and July 2024. An interview guide was developed based on the literature review and included the following questions:

(1) Can you describe your sitting habits and daily activities during a typical day?
(2) What physical changes or discomfort (e.g., fatigue, muscle soreness, low back pain, or back pain) do you usually experience after prolonged sitting?
(3) Are you aware of the potential health risks associated with prolonged sedentary behavior?
(4) Have you heard of “sarcopenia” or “muscle mass loss”? What do you know about these conditions?
(5) In your opinion, what symptoms may indicate declining muscle function?
(6) Through which channels would you prefer to receive information or support to reduce sedentary time and increase physical activity?
(7) If a tool were available to assess the risk of sedentary behavior-related impairment in muscle health, what domains do you think it should include?

During the same period, pilot interviews were conducted with three experts using the following interview guide:

(1) What are the earliest and most characteristic manifestations of pre-sarcopenia among sedentary young adults?
(2) Which behavioral characteristics are most important when assessing the risks associated with sedentary behavior?
(3) In the absence of objective measurements of muscle mass, which self-reported symptoms or simple functional assessments are most sensitive for detecting early declines in muscle function?
(4) Is the dimension of “intervention intention” necessary? What is its value for early screening and subsequent management?
(5) What factors should be emphasized to ensure the feasibility and practicality of the scale?

Subsequently, semi-structured interviews were conducted with 12 young adults with T2DM who engaged in prolonged sedentary behavior and 10 experts. Audio recordings were transcribed, verified, and organized within 24 h after each interview. The transcripts were analyzed using qualitative content analysis, with themes identified through repeated coding, comparison, and categorization. Findings from the literature review, qualitative interviews, and research team discussions were integrated to develop a preliminary scale comprising of four dimensions: behavioral risk perception, muscle function monitoring, sedentary behavior characteristics, and intervention intention with a total of 17 items.

##### 2.2.2.4 Expert correspondence consultation

The Delphi questionnaires were distributed electronically via email. The response rate was 100% for both rounds of consultation, and the expert authority coefficients (Cr) were 0.87 and 0.89, respectively. During the first round, the importance of each item was rated by the experts using a 5-point Likert scale, and recommendations for revision were provided. Items with a mean importance score > 4.0 and a coefficient of variation < 0.25 were retained. Based on expert feedback and research team discussions, 6 items were deleted, 9 items were revised or merged, and 2 new items were added. Following the second round, consensus among the experts was achieved, and all items met the predefined criteria. The preliminary scale comprised of 19 items across 4 dimensions: behavioral risk perception, muscle function monitoring, sedentary behavior characteristics, and intervention intention.

##### 2.2.2.5 Pilot survey

A pilot study was conducted among 30 young adults with T2DM who engaged in prolonged sedentary behavior in October 2024 using a self-developed general information questionnaire and the preliminary version of the scale. After written informed consent had been obtained, paper questionnaires were administered. Participants were invited to provide feedback regarding the clarity, comprehensibility, and appropriateness of each item and its response options to evaluate the readability and applicability of the scale. Several participants indicated that the wording of some items was ambiguous or difficult to understand. Based on this feedback, two items were revised by the research team:

(1) “I can perceive changes in my muscle function” was revised to “I can detect changes in my muscle strength or endurance.”
(2) “I will take positive actions to improve my muscle health” was revised to “I am willing to try strength training to improve my muscle function.”

These revisions improved the clarity of the items and enhanced their content validity.

##### 2.2.2.6 Formal survey

The questionnaire consisted of two sections. The first section collected demographic and clinical data, including sex, age, body mass index, daily sedentary time, and occupation. The second section comprised the preliminary version of the Pre-sarcopenia Risk Assessment Scale for Sedentary Young Adults with T2DM. Each item was rated on a 5-point Likert scale, with response options ranging from 1 (“Never”) to 5 (“Always”). For the preliminary 19-item version, total scores ranged from 19 to 95. After Item 11 was removed, the final 18-item version was scored as follows: total scores range from 18 to 90; no item is reverse-scored in the final version; for missing responses, if ≤20% of items are missing, the missing item is replaced by the mean score of completed items in the same dimension; questionnaires with >20% missing data are excluded. Higher total and dimension scores indicate greater pre-sarcopenia-related behavioral and functional risk perception. No clinical cut-off is currently recommended, and scores should be interpreted as continuous risk indicators. Before the formal survey, all investigators received standardized training regarding the study procedures and questionnaire administration.

Data were collected using both online and paper-based questionnaires, and standardized instructions were provided to all participants. A total of 215 valid questionnaires were collected during the first phase of the survey and used for item analysis and EFA. Subsequently, 220 valid questionnaires were collected and used for CFA and psychometric evaluation of the scale during the second phase.

#### 2.2.3 Statistical methods

Data were analyzed using SPSS version 22.0 and AMOS version 20.0. Item analysis was conducted to assess item quality using the critical ratio (CR) method, item-total correlation analysis, and reliability analysis. The psychometric properties of the scale were evaluated by assessing its validity and reliability. Content validity was examined using the content validity index (CVI) based on expert ratings obtained during the Delphi consultation. Construct validity was evaluated in two stages. First, EFA was conducted using data from 215 participants (EFA group) to identify the underlying factor structure. Subsequently, CFA was performed using data from an independent sample of 220 participants (CFA group) to assess the fit of the proposed measurement model. In the CFA model, the four latent factors were allowed to correlate to examine the relationships among the dimensions. Reliability was assessed by examining internal consistency, split-half reliability, and test–retest reliability. To evaluate the temporal stability of the scale, 50 participants were randomly selected from the CFA group and asked to complete the scale again 2 weeks after the initial assessment. The intraclass correlation coefficient (ICC) was calculated to assess test–retest reliability. A two-sided *p*-value <0.05 was considered statistically significant. CFA was specified with correlated latent factors because the four dimensions were conceptually related. The correlations among latent factors were examined to evaluate whether the dimensions were empirically distinct.

## 3 Results

### 3.1 Results of item analysis

Item analysis of the preliminary scale was conducted using multiple screening methods.

(1) CR analysis: Participants were classified into high-score and low-score groups according to the upper and lower 27% of the total scale scores. Independent-samples *t*-tests were performed to calculate the CR for each item.

The results demonstrated that Item 11 (“I think my muscle function is normal”) had a CR value of 2.91 (*p* > 0.05), whereas the CR values for the remaining items ranged from 5.24 to 20.16 (all *p* < 0.001). All items except Item 11 met the predefined criterion of CR > 3.00.

(2) Item-total correlation analysis: Pearson correlation coefficients between individual item scores and the total scale score for the 18 retained items ranged from 0.593 to 0.882. Item 11 had an item-total correlation coefficient of 0.358, which was below the predefined criterion of 0.40.
(3) Reliability analysis: Deletion of Item 11 increased the Cronbach’s α coefficient of the overall scale from 0.891 to 0.894.

Based on the results of the CR analysis, item-total correlation analysis, and reliability analysis, Item 11 (“I think my muscle function is normal”) was removed. The remaining items were renumbered, resulting in a final set of 18 items for subsequent factor analyses.

### 3.2 Results of validity analysis

#### 3.2.1 Exploratory factor analysis

The suitability of the data for EFA was assessed using the responses from 215 participants. The Kaiser–Meyer–Olkin measure of sampling adequacy was 0.928, and Bartlett’s test of sphericity was statistically significant (*χ*^2^ = 2987.52, *p* < 0.001), indicating that the data were suitable for factor analysis.

Principal component analysis (PCA) with varimax orthogonal rotation was used as an initial exploratory dimension-reduction method. PCA was selected to identify empirical item clusters and reduce dimensionality; it was not intended to test a latent common-factor model. Because the four proposed dimensions were conceptually related, CFA was subsequently specified with correlated latent factors, and the latent factor correlations were examined. Following iterative analyses guided by the theoretical framework, four factors with eigenvalues greater than 1 were retained. These four factors accounted for a cumulative explained variance of 81.25%.

The factor loadings ranged from 0.58 to 0.86 on the corresponding factors, and no cross-loading exceeded 0.40. These findings indicated a clear four-factor structure, with the item distribution consistent with the predefined theoretical dimensions. The detailed results are presented in Table 2.

**Table 2:** Factor loadings for the pre-sarcopenia risk assessment scale for sedentary young adults with T2DM.

Items	F1	F2	F3	F4
F1				
I can detect changes in my muscle strength.	0.82	0.12	0.09	0.05
I worry that sedentary behavior will exert adverse impacts on my muscle health.	0.78	0.21	0.13	−0.04
I think it is necessary to take action to improve muscle function at present.	0.75	0.18	0.25	0.11
I realize that declining muscle function will affect my long-term health.	0.71	0.10	0.15	0.18
I understand that sedentary behavior is a major risk factor for muscle health problems.	0.68	0.23	0.08	0.12
F2				
My prolonged sitting time in daily life is rarely interrupted.	0.17	0.84	0.08	0.06
My work or study requires me to maintain a sitting posture for a long time.	0.09	0.81	0.12	0.13
Most of my leisure activities are sedentary (e.g., scrolling mobile phones, surfing the Internet).	0.22	0.79	0.07	0.02
I usually stay seated for more than 1 h before standing up and moving around.	0.15	0.76	0.10	0.09
My travel mainly relies on vehicles rather than walking or cycling.	0.11	0.73	0.14	0.05
F3				
I feel leg fatigue when walking upstairs.	0.11	0.05	0.86	0.12
I have difficulty standing up from a chair.	0.08	0.13	0.83	0.09
I have experienced increased overall fatigue recently.	0.23	0.10	0.80	0.14
I feel that it is more strenuous than before when carrying heavy objects.	0.14	0.09	0.77	0.11
F4				
I am willing to reduce my daily sedentary time.	0.13	0.06	0.11	0.85
I plan to increase my daily physical activity volume.	0.10	0.15	0.09	0.82
I am willing to try strength training to improve my muscle function.	0.21	0.04	0.12	0.78
I will take the initiative to learn methods for improving muscle health.	0.19	0.08	0.10	0.74
Eigenvalue	5.12	4.85	4.20	3.58
Variance contribution rate (%)	24.18	20.76	18.95	17.36
Cumulative variance contribution rate (%)	24.18	44.94	63.89	81.25

F1, Behavioral risk perception; F2, Sedentary behavior characteristics; F3, Muscle function monitoring; F4, Intervention intention.

#### 3.2.2 Confirmatory factor analysis

CFA demonstrated a satisfactory model fit. The *χ*^2^/df ratio was 1.583, the root mean square error of approximation was 0.076 (90% confidence interval: 0.055–0.097), the standardized root mean square residual was 0.038, the normed fit index was 0.923, the comparative fit index was 0.965, and the goodness-of-fit index was 0.917. The standardized factor loadings ranged from 0.56 to 0.83, and all factor loadings were statistically significant (all *p* < 0.001). The standardized correlations among the four latent factors ranged from 0.34 to 0.62, indicating that the dimensions were related but not redundant.

#### 3.2.3 Content validity

The expert evaluation indicated excellent content validity. The scale-level average content validity index (S-CVI/Ave) was 0.92, while the item-level content validity index (I-CVI) ranged from 0.87 to 1.00 across all items.

#### 3.2.4 Results of reliability analysis

The Cronbach’s α coefficient for the overall scale was 0.894. The Cronbach’s α coefficients for the four dimensions were 0.872 for behavioral risk perception, 0.861 for muscle function monitoring, 0.836 for sedentary behavior characteristics, and 0.819 for intervention intention. The split-half reliability coefficient was 0.792, and the test–retest reliability, assessed over a 2-week interval, yielded an ICC of 0.843.

#### 3.2.5 Exploratory correlations between scale scores and objective functional measures

Exploratory correlation analyses were conducted to examine the relationships between scale scores and objective functional measures. The total scale score was negatively correlated with handgrip strength (*r* = −0.35, *p* < 0.001) and positively correlated with the five-repetition sit-to-stand test (*r* = 0.30, *p* < 0.001). Among the four dimensions, the behavioral risk perception score was correlated with handgrip strength (*r* = −0.28, *p* < 0.001) and the five-repetition sit-to-stand test (*r* = 0.24, *p* < 0.001); the sedentary behavior characteristics score was correlated with handgrip strength (*r* = −0.26, *p* < 0.001) and the five-repetition sit-to-stand test (*r* = 0.22, *p* < 0.001); the muscle function monitoring score was correlated with handgrip strength (*r* = −0.24, *p* < 0.001) and the five-repetition sit-to-stand test (*r* = 0.21, *p* < 0.001); and the intervention intention score was correlated with handgrip strength (*r* = −0.21, *p* < 0.001) and the five-repetition sit-to-stand test (*r* = 0.18, *p* = 0.002). These analyses were exploratory and do not establish full criterion validity (Table 3).

**Table 3:** Correlations between scale scores and objective functional measures.

Variable	Handgrip strength (kg)	Five-repetition sit-to-stand test (s)
Total scale score	*r* = −0.35, *p* < 0.001	*r* = 0.30, *p* < 0.001
Behavioral risk perception	*r* = −0.28, *p* < 0.001	*r* = 0.24, *p* < 0.001
Sedentary behavior characteristics	*r* = −0.26, *p* < 0.001	*r* = 0.22, *p* < 0.001
Muscle function monitoring	*r* = −0.24, *p* < 0.001	*r* = 0.21, *p* < 0.001
Intervention intention	*r* = −0.21, *p* < 0.001	*r* = 0.18, *p* = 0.002

## 4 Discussion

### 4.1 Scientific nature of the development of the pre-sarcopenia risk assessment scale

This study was conducted in accordance with the COSMIN guidelines for the development of health measurement instruments and was guided by the HBM and the conceptual framework linking behavioral risk factors with muscle function. This theoretical foundation provided a systematic basis for item generation and scale development. During the item development phase, multiple approaches, including a comprehensive literature review, semi-structured interviews, and Delphi consultation, were integrated to ensure comprehensive coverage of the key constructs associated with sedentary behavior and pre-sarcopenia. The Delphi panel comprised of experts in sports medicine, rehabilitation nursing, public health, and related disciplines from tertiary hospitals and universities across multiple provinces and municipalities in China. The response rate was 100% for both rounds of the Delphi consultation, and the expert authority coefficients exceeded 0.80 in both rounds, indicating the credibility and scientific rigor of the content development process.

A pilot study involving 30 young adults with T2DM who engaged in prolonged sedentary behavior was conducted to assess the clarity, comprehensibility, and acceptability of the preliminary scale. Based on the feedback of the participants, the wording of selected items was refined to improve readability and to better reflect the language and cognitive characteristics of the target population, thereby further enhancing the content validity and practical applicability of the scale.

### 4.2 Reliability and validity of the pre-sarcopenia risk assessment scale for sedentary young adults with T2DM

The findings of the psychometric evaluation indicated that the newly developed scale possesses satisfactory preliminary reliability and construct validity. Item analysis demonstrated that the CR values of the retained items ranged from 5.24 to 20.16, while the item-total correlation coefficients of the retained items ranged from 0.593 to 0.882. All retained items met the predefined psychometric criteria (CR > 3.0 and r > 0.40), indicating satisfactory item discrimination and internal homogeneity. EFA identified four common factors, which collectively explained 81.25% of the total variance. The four-factor structure was subsequently confirmed by CFA, with all model fit indices meeting the recommended criteria, thereby supporting the construct validity of the scale. The moderate correlations among the four latent factors (range, 0.34–0.62) further supported the construct validity of the scale, suggesting that the dimensions were conceptually related but empirically distinguishable. Regarding content validity, the S-CVI/Ave was 0.92, and the I-CVI ranged from 0.87 to 1.00.

These findings indicate that the scale items adequately represent the principal characteristics associated with the risk of pre-sarcopenia in young adults with T2DM who engage in prolonged sedentary behavior. Reliability analysis demonstrated satisfactory measurement properties. The overall Cronbach’s α coefficient was 0.894, indicating good internal consistency, while the split-half reliability coefficient was 0.792, supporting the internal stability of the scale. The test–retest reliability coefficient was 0.843, indicating good temporal stability over a 2-week interval.

### 4.3 Applicability of the pre-sarcopenia risk assessment scale for sedentary young adults with T2DM

The developed scale may serve as a practical preliminary risk-assessment instrument for identifying individuals with heightened pre-sarcopenia-related behavioral and functional risk among young adults with T2DM who engage in prolonged sedentary behavior, thereby providing a basis for further objective assessment and targeted preventive interventions. Prolonged sedentary behavior is a common lifestyle characteristic of this population. In the presence of the metabolic abnormalities associated with T2DM, sedentary behavior may further accelerate the decline in muscle mass and muscle function through multiple pathophysiological mechanisms, including the inhibition of muscle protein synthesis, reduced insulin sensitivity, and chronic low-grade inflammation (17–19).

Consequently, young adults with T2DM who engage in prolonged sedentary behavior constitute a population at increased risk of pre-sarcopenia. Early impairments in muscle function resulting from the combined effects of prolonged sedentary behavior and metabolic abnormalities are often subtle and potentially reversible unlike age-related sarcopenia, providing an important opportunity for early identification and timely intervention (20, 21). By facilitating preliminary risk identification, the present scale may support further objective assessment and risk stratification, and guide the implementation of targeted interventions aimed at improving muscle health and preventing progression to sarcopenia.

Regarding assessment instruments, currently available screening methods have several limitations. Internationally recognized screening tools, such as the SARC-F questionnaire, were developed primarily for older adults and include items focusing on age-related functional impairments, such as a history of falls and difficulty walking. Consequently, these instruments have limited sensitivity for detecting early declines in muscle function associated with prolonged sedentary behavior and metabolic abnormalities in young adults with T2DM (22, 23).

Although imaging- and device-based diagnostic methods for sarcopenia, such as dual-energy X-ray absorptiometry and bioelectrical impedance analysis, provide objective measurements of muscle mass, their application is constrained by limited equipment availability, operational complexity, and high costs, reducing their feasibility for large-scale screening in outpatient and community settings (24, 25).

The scale developed in this study provides a simple, self-administered preliminary risk-assessment instrument that is practical for routine use. Compared with general physical activity assessment tools, such as the International Physical Activity Questionnaire, this scale was specifically developed for young adults with T2DM who engage in prolonged sedentary behavior (26, 27). It incorporates four dimensions: behavioral risk perception, muscle function monitoring, sedentary behavior characteristics, and intervention intention to provide a comprehensive assessment of behavioral risk, muscle function, and health-related behavioral intentions. By integrating these dimensions, the scale captures the distinctive behavioral and cognitive characteristics associated with the risk of pre-sarcopenia in this population.

The scale also demonstrates potential for clinical and research applications. In clinical settings, it may facilitate preliminary risk assessment during outpatient visits, enabling healthcare professionals to identify individuals who may require further objective evaluation and to provide timely, individualized health education and intervention recommendations. In community settings, the scale may be used as a preliminary risk-assessment instrument to support primary healthcare providers in identifying individuals with heightened risk and implementing early risk stratification and management. In research settings, the scale provides a standardized instrument for investigating the associations between sedentary behavior, glycometabolic status, and changes in muscle function, while also serving as a preliminary outcome measure for evaluating the effectiveness of behavioral interventions and clinical management strategies. Because no clinical cut-off has been established, total and dimension scores should be interpreted as continuous indicators of pre-sarcopenia-related behavioral and functional risk perception rather than as diagnostic classifications. Higher scores indicate a greater need for further objective assessment.

### 4.4 Limitations

This study has several limitations. First, participants were recruited by convenience sampling from a single hospital, which may limit the external validity of the findings; external validation in independent, multicenter populations is needed.

Second, criterion-related validity against objective measures of muscle mass, muscle strength, and physical performance was not established. Although handgrip strength and the five-repetition sit-to-stand test were collected as baseline functional indicators, muscle mass was not measured, and no accepted reference standard for pre-sarcopenia was applied. Third, no confirmed pre-sarcopenia case cohort was included; therefore, no clinical cut-off, sensitivity, specificity, or ROC analysis could be validly established. Fourth, PCA with varimax rotation was used as an initial exploratory method; future studies should compare common-factor extraction and oblique rotation. Therefore, the scale should be regarded as a preliminary risk-assessment instrument requiring further external and criterion-based validation.

## 5 Conclusion

This study developed a pre-sarcopenia risk assessment scale comprising 4 dimensions and 18 items for young adults with T2DM who engage in prolonged sedentary behavior. The findings indicated that the scale has satisfactory preliminary reliability and construct validity, supporting its use as a preliminary self-reported risk-assessment instrument for pre-sarcopenia-related behavioral and functional risk in this population. The scale may facilitate preliminary risk identification and provide a basis for further objective assessment and targeted health management strategies in clinical and community settings. No clinical cut-off is currently recommended.

Future studies should further assess the generalizability and applicability of the scale among young adults with T2DM who engage in prolonged sedentary behavior across diverse geographic regions and healthcare settings. Future studies should include objective measures of muscle mass, muscle strength, and physical performance and confirmed diagnostic criteria to establish criterion-related validity, optimal cut-off values, sensitivity, specificity, and predictive values. Prospective studies are also warranted to evaluate the utility of the scale in guiding early behavioral and metabolic interventions and to determine its effectiveness in improving muscle health and diabetes-related outcomes.

## Data Availability

The raw data supporting the conclusions of this article will be made available by the authors, without undue reservation.
